# Supplementary material for: Burden of leprosy and associated risk factors for disabilities in Tanzania from 2017 to 2020
Source: PLoS One. 2024 Oct 10;19(10):e0311676. doi: 10.1371/journal.pone.0311676 (PMC11466380; doi:10.1371/journal.pone.0311676)
Supplement: S1 Table — (PDF) [file pone.0311676.s001.pdf]

S1 Table. Trend in Leprosy elimination indicators by regions from the year 2017 to 2020.

Regions	Year 2017				Year 2018				Year 2019				Year 2020			
	Point prevalence per 10,000 population	New cases detected	% Children in new cases	% G2D in new cases	Point prevalence per 10,000 population	New cases detected	% Children in new cases	% G2D in new cases	Point prevalence per 10,000 population	New cases detected	% Children in new cases	% G2D in new cases	Point prevalence per 10,000 population	New cases detected	% Children in new cases	% G2D in new cases
**Arusha**	***				0.01	2	0.0	0.0	0.01	3	0.0	0.0	0.00	1	0.0	0.0
**Dar Es Salaam**	0.30	153	2.6	10.5	0.47	231	2.2	5.6	0.38	177	4.0	10.7	0.28	138	2.2	10.1
**Dodoma**	0.42	93	4.3	5.4	0.35	86	5.8	3.5	0.22	57	0.0	7.0	0.17	45	0.0	8.9
**Geita**	0.37	73	1.4	2.7	0.58	128	2.3	3.9	0.48	112	2.7	11.6	0.24	58	1.7	6.9
**Iringa**	0.08	7	0.0	42.9	0.09	10	0.0	30.0	0.06	7	0.0	14.3	0.08	8	0.0	37.5
**Kagera**	0.08	21	0.0	38.1	0.07	18	5.6	22.2	0.09	21	0.0	28.6	0.06	14	0.0	21.4
**Katavi**	0.24	16	0.0	0.0	0.99	73	4.1	24.7	0.97	74	0.0	8.1	0.74	60	1.7	1.7
**Kigoma**	0.42	100	5.0	7.0	0.37	94	5.3	17.0	0.37	90	1.1	13.3	0.32	89	2.2	12.4
**Kilimanjaro**	0.02	4	0.0	0.0	0.03	6	16.7	33.3	0.03	4	0.0	0.0	0.01	2	0.0	0.0
**Lindi**	1.35	120	3.3	6.7	1.29	126	1.6	8.7	1.31	124	3.2	8.9	0.85	81	3.7	4.9
**Manyara**	0.02	2	0.0	50.0	0.02	3	0.0	33.3	0.01	2	0.0	0.0	***			
**Mara**	0.02	4	0.0	0.0	0.06	10	0.0	10.0	0.08	19	0.0	15.8	0.05	13	0.0	0.0
**Mbeya**	0.12	23	0.0	8.7	0.12	23	8.7	17.4	0.08	16	6.3	18.8	0.02	2	0.0	0.0
**Morogoro**	0.62	153	2.0	1.3	1.06	272	4.4	1.1	1.09	283	4.6	3.9	0.95	240	3.3	5.4
**Mtwara**	0.79	106	3.8	3.8	0.74	101	2.0	9.9	0.61	85	0.0	11.8	0.50	73	0.0	2.7
**Mwanza**	0.18	51	2.0	19.6	0.22	67	1.5	14.9	0.25	80	1.3	12.5	0.09	33	3.0	27.3
**Njombe**	0.05	4	0.0	25.0	0.11	8	0.0	37.5	0.01	1	0.0	0.0	0.02	1	0.0	100.0
**Pemba**	0.37	16	25.0	0.0	0.15	8	0.0	0.0	0.55	27	3.7	22.2	0.39	21	4.8	23.8
**Pwani**	0.74	89	6.7	12.4	0.52	65	6.2	7.7	0.56	65	0.0	10.8	0.58	75	6.7	2.7
**Rukwa**	0.98	114	3.5	1.8	0.89	106	0.0	1.9	0.90	110	3.6	2.7	0.48	60	5.0	1.7
**Ruvuma**	0.59	86	1.2	2.3	0.44	70	1.4	0.0	0.43	68	1.5	0.0	0.37	56	1.8	7.1
**Shinyanga**	0.33	56	1.8	42.9	0.22	39	0.0	33.3	0.28	52	0.0	21.2	0.14	26	3.8	15.4
**Simiyu**	***				0.02	4	25.0	0.0	0.04	8	0.0	0.0	0.01	2	0.0	0.0
**Singida**	0.12	18	5.6	0.0	0.12	20	0.0	10.0	0.12	20	0.0	5.0	0.05	7	0.0	28.6
**Songwe**	0.03	3	0.0	0.0	0.05	6	0.0	33.3	0.07	7	0.0	28.6	0.01	1	0.0	0.0
**Tabora**	0.26	61	4.9	11.5	0.24	67	3.0	20.9	0.23	59	5.1	16.9	0.25	73	0.0	23.3
**Tanga**	0.64	142	3.5	12.0	0.65	147	6.8	12.2	0.49	116	5.2	7.8	0.41	96	3.1	18.8
**Unguja**	0.68	70	20.0	1.4	0.75	79	17.7	5.1	1.31	136	19.9	5.1	1.01	109	20.2	3.7

***** No case registered for the whole year**
