# Supplementary figures and images for: Burden of leprosy and associated risk factors for disabilities in Tanzania from 2017 to 2020
Source: PLoS One. 2024 Oct 10;19(10):e0311676. doi: 10.1371/journal.pone.0311676 (PMC11466380; doi:10.1371/journal.pone.0311676)

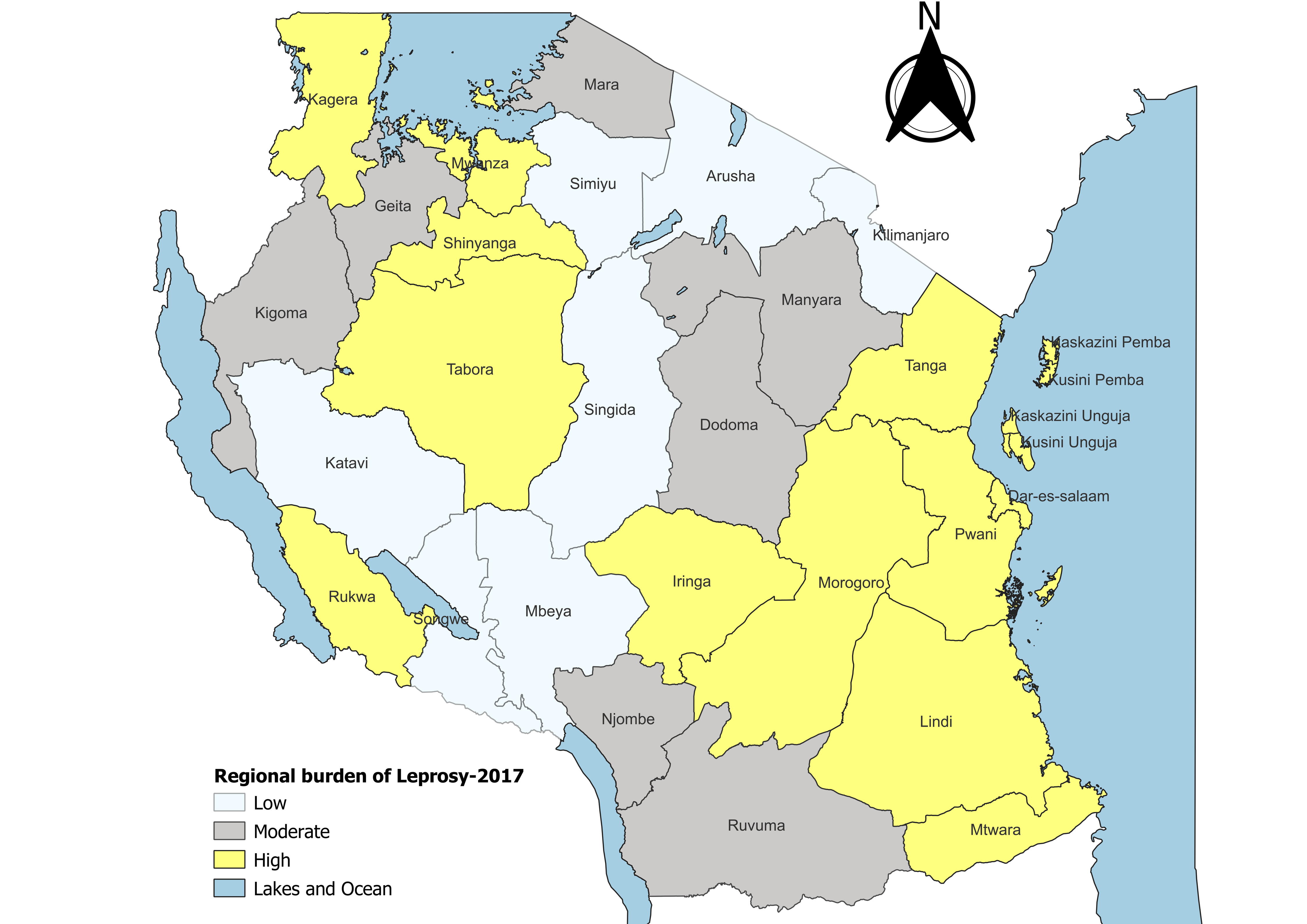

Supplement: S1 Fig — Map was drawn using QGIS desktop software 3.26.3. The shapefiles used were from an openly available source (https://www.nbs.go.tz/statistics/topic/gis) The shapefiles were made based on the 2012 population and housing census, but in this study, shapefiles have been modified to capture all the regions information. (TIF) [file pone.0311676.s002.tif]

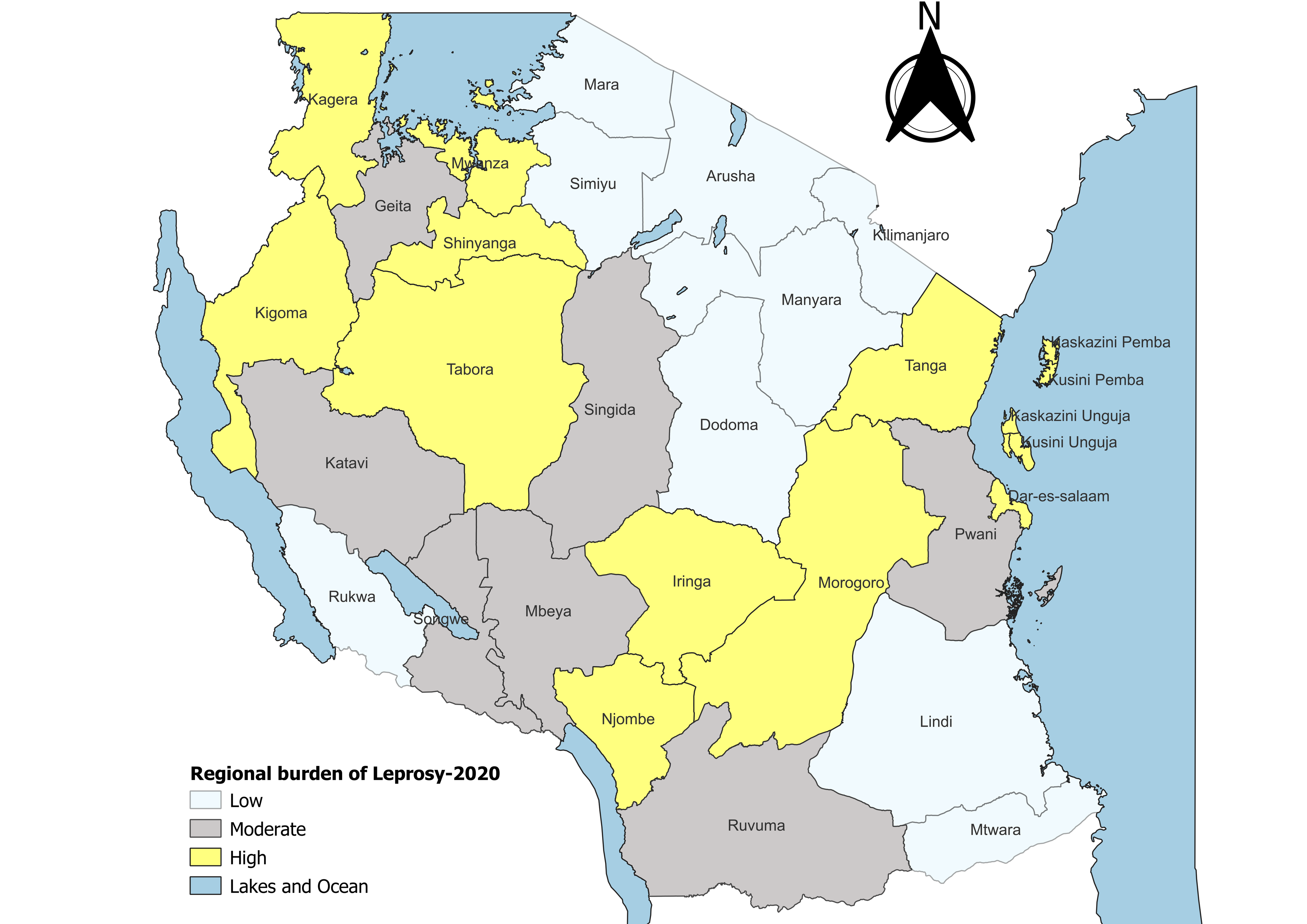

Supplement: S2 Fig — Map was drawn using QGIS desktop software 3.26.3. The shapefiles used were from an openly available source (https://www.nbs.go.tz/statistics/topic/gis). The shapefiles were made based on the 2012 population and housing census, but in this study, shapefiles have been modified to capture all the regions information. (TIF) [file pone.0311676.s003.tif]
