# Supplementary material for: Burden of leprosy and associated risk factors for disabilities in Tanzania from 2017 to 2020
Source: PLoS One. 2024 Oct 10;19(10):e0311676. doi: 10.1371/journal.pone.0311676 (PMC11466380; doi:10.1371/journal.pone.0311676)
Supplement: Attachment — Submitted filename: PONE-D-23-30254 (1).pdf [file pone.0311676.s005.pdf]

PLOS ONE

# Burden of Leprosy and associated risk factors for disabilities in Tanzania from 2017 to 2020: Analytical cross section study

--Manuscript Draft--

| | |
|---|---|
| **Manuscript Number:** | PONE-D-23-30254 |
| **Article Type:** | Research Article |
| **Full Title:** | Burden of Leprosy and associated risk factors for disabilities in Tanzania from 2017 to 2020: Analytical cross section study |
| **Short Title:** | Burden of Leprosy and associated risk factors for disabilities in Tanzania from 2017 to 2020 |
| **Corresponding Author:** | George Mrema, MD, MSc<br>Ministry of Health and Community Service: Ministry of Health Community Development Gender Elderly and Children<br>DODOMA, TANZANIA, UNITED REPUBLIC OF |
| **Keywords:** | Leprosy, burden, disability |
| **Abstract:** | Background: Leprosy is caused by mycobacterium leprae which affects skin, nerves, eyes and nasal mucosa. Despite global elimination progress, Tanzania remains among 13 countries reporting more than 1000 leprosy cases annually. In 2021, Tanzania detected 1,511 new cases with 10% having grade II disability. Additionally, 14 councils in the country had leprosy rates above 10 cases per 100,000 population. Hence this study aimed at assessing the burden of leprosy and associated factors for disabilities in Tanzania from 2017 to 2020.<br>Methodology: Analytical cross-sectional study was used to investigate all registered treated Leprosy patients from 2017 to 2020. To assess disease burden and risk factor for disability, Leprosy Burden Score (LBS) and logistic regression were used respectively.<br>Result: A total of 6963 leprosy cases were found from 2017 to 2020. Between the years 2017 and 2020, point prevalence new case detection rate remained the same over the study period. Independent risk factors for disability among leprosy cases included male sex (Adjusted Odds Ratio (AOR)=1.45, 95% Confidence Interval (CI) 1.29-1.63), patient age above 55 years (AOR=2.79, 95% CI 1.95-4.01), Multibacillary (MB) leprosy (AOR=1.46, 95% CI 1.15-1.84), previous treatment (AOR=2.11 95% CI 1.65-2.69) and positive Human Immunodeficiency Virus (HIV) status (AOR=1.67 95% CI 1.16-2.41).<br>Conclusion: No significant change in point prevalence and new case detection rate in the country was observed over the past four years. However, various risk factors including age, gender, patient category, type of leprosy and HIV status were found to be associated with leprosy disability. To combat this, strategies and interventions are needed such as active case finding, post-exposure prophylaxis, provision of MDT and engaging political leaders and key actors in community sensitization, with a focus on endemic areas. |
| **Order of Authors:** | George Mrema, MD, MSc |
| | Ally Hussein, MD,MSc |
| | Welema Magoge, BSc.EHS, MSc |
| | Vida Mmbaga, MD,MSc |
| | Azma Simba, MD, MSc |
| | Robert Balama |
| | Emmanuel Nkiligi |
| | Paul Shunda |
| | Deus Kamara |
| | Riziki Kisonga |
| | |

Powered by Editorial Manager® and ProduXion Manager® from Aries Systems Corporation

| | Gideon Kwesigabo |
|---|---|
| **Additional Information:** | |
| **Question** | **Response** |
| **Financial Disclosure**<br><br>Enter a financial disclosure statement that describes the sources of funding for the work included in this submission. Review the submission guidelines for detailed requirements. View published research articles from *PLOS ONE* for specific examples.<br><br>This statement is required for submission and **will appear in the published article** if the submission is accepted. Please make sure it is accurate.<br><br>**Unfunded studies**<br>Enter: *The author(s) received no specific funding for this work.*<br><br>**Funded studies**<br>Enter a statement with the following details:<br>• Initials of the authors who received each award<br>• Grant numbers awarded to each author<br>• The full name of each funder<br>• URL of each funder website<br>• Did the sponsors or funders play any role in the study design, data collection and analysis, decision to publish, or preparation of the manuscript?<br>• **NO** - Include this sentence at the end of your statement: *The funders had no role in study design, data collection and analysis, decision to publish, or preparation of the manuscript.*<br>• **YES** - Specify the role(s) played.<br><br>* typeset | The author(s) received no specific funding for this work |
| **Competing Interests**<br><br>Use the instructions below to enter a competing interest statement for this submission. On behalf of all authors, disclose any competing interests that could be perceived to bias this work—acknowledging all financial support and any other relevant financial or non-financial competing interests. | The authors have declared that no competing interests exist |

Powered by Editorial Manager® and ProduXion Manager® from Aries Systems Corporation

| | |
|---|---|
| This statement is required for submission and **will appear in the published article** if the submission is accepted. Please make sure it is accurate and that any funding sources listed in your Funding Information later in the submission form are also declared in your Financial Disclosure statement.<br><br>View published research articles from *PLOS ONE* for specific examples.<br><br>**NO authors have competing interests**<br><br>Enter: *The authors have declared that no competing interests exist.*<br><br>**Authors with competing interests**<br><br>Enter competing interest details beginning with this statement:<br><br>*I have read the journal's policy and the authors of this manuscript have the following competing interests: [insert competing interests here]*<br><br>* typeset | |
| **Ethics Statement**<br><br>Enter an ethics statement for this submission. This statement is required if the study involved:<br><br>• Human participants<br>• Human specimens or tissue<br>• Vertebrate animals or cephalopods<br>• Vertebrate embryos or tissues<br>• Field research<br><br>Write "N/A" if the submission does not require an ethics statement.<br><br>General guidance is provided below. Consult the submission guidelines for detailed instructions. **Make sure that all** | The study reviewed secondary data that are part of routine public health practices. Furthermore, the data analyzed in the study did not include patient identifiers but instead we utilized distinct identification codes to ensure the protection of individuals' privacy. Lastly, approval to use the national TB program data was obtained from Permanent Secretary at the Ministry of Health |

Powered by Editorial Manager® and ProduXion Manager® from Aries Systems Corporation

| | |
|---|---|
| **information entered here is included in the Methods section of the manuscript.**<br><br>**Format for specific study types**<br><br>**Human Subject Research (involving human participants and/or tissue)**<br>• Give the name of the institutional review board or ethics committee that approved the study<br>• Include the approval number and/or a statement indicating approval of this research<br>• Indicate the form of consent obtained (written/oral) or the reason that consent was not obtained (e.g. the data were analyzed anonymously)<br><br>**Animal Research (involving vertebrate animals, embryos or tissues)**<br>• Provide the name of the Institutional Animal Care and Use Committee (IACUC) or other relevant ethics board that reviewed the study protocol, and indicate whether they approved this research or granted a formal waiver of ethical approval<br>• Include an approval number if one was obtained<br>• If the study involved *non-human primates*, add *additional details* about animal welfare and steps taken to ameliorate suffering<br>• If anesthesia, euthanasia, or any kind of animal sacrifice is part of the study, include briefly which substances and/or methods were applied<br><br>**Field Research**<br><br>Include the following details if this study involves the collection of plant, animal, or other materials from a natural setting:<br>• Field permit number<br>• Name of the institution or relevant body that granted permission | |
| **Data Availability**<br><br>Authors are required to make all data underlying the findings described fully available, without restriction, and from the time of publication. PLOS allows rare exceptions to address legal and ethical | No - some restrictions will apply |

| | |
|---|---|
| concerns. See the PLOS Data Policy and FAQ for detailed information.<br><br>A Data Availability Statement describing where the data can be found is required at submission. Your answers to this question constitute the Data Availability Statement and **will be published in the article**, if accepted.<br><br>**Important:** Stating 'data available on request from the author' is not sufficient. If your data are only available upon request, select 'No' for the first question and explain your exceptional situation in the text box.<br><br>Do the authors confirm that all data underlying the findings described in their manuscript are fully available without restriction? | |
| **Describe where the data may be found in full sentences. If you are copying our sample text, replace any instances of XXX with the appropriate details.**<br><br>• If the data are **held or will be held in a public repository**, include URLs, accession numbers or DOIs. If this information will only be available after acceptance, indicate this by ticking the box below. For example: *All XXX files are available from the XXX database (accession number(s) XXX, XXX.*).<br>• If the data are all contained **within the manuscript and/or Supporting Information files**, enter the following: *All relevant data are within the manuscript and its Supporting Information files.*<br>• If neither of these applies but you are able to provide **details of access elsewhere**, with or without limitations, please do so. For example:<br><br>*Data cannot be shared publicly because of [XXX]. Data are available from the XXX Institutional Data Access / Ethics Committee (contact via XXX) for researchers who meet the criteria for access to confidential data.* | The data underlying the results presented in the study are available from Tanzania National Tuberculosis and Leprosy Program can be reached via info@ntlp.go.tz |

Powered by Editorial Manager® and ProduXion Manager® from Aries Systems Corporation

| | |
|---|---|
| *The data underlying the results presented in the study are available from (include the name of the third party and contact information or URL).*<br>• This text is appropriate if the data are owned by a third party and authors do not have permission to share the data.<br><br>* typeset | |
| Additional data availability information: | |

Powered by Editorial Manager® and ProduXion Manager® from Aries Systems Corporation

Manuscript

Click here to access/download;Manuscript;Manuscript-Leprosy Final George Mrema.docx

**Burden of Leprosy and associated risk factors for disabilities in Tanzania from 2017 to 2020**

George Mrema[1,2]*, Ally Hussein[1,2], Welema Magoge[3], Vida Mmbaga[3], Azma Simba[3], Robert Balama[4], Emmanuel Nkiligi[4], Paul Shunda[4], Deus Kamara[4], Riziki Kisonga[4], Gideon Kwesigabo[1]

[1]Department of Epidemiology and Biostatistics, Muhimbili University of Health and Allied Sciences, Dar es Salaam, Tanzania

[2]Tanzania Field Epidemiology and Laboratory Training Program, Dar es Salaam, Tanzania

[3]Ministry of Health, Epidemiology and Disease Control Section, Dodoma, Tanzania

[4]Ministry of Health, National Tuberculosis and Leprosy Program, Dodoma, Tanzania

*Email: drgeorgemrema@gmail.com (GM)

# Abstract

**Background:** Leprosy is caused by mycobacterium leprae which affects skin, nerves, eyes and nasal mucosa. Despite global elimination progress, Tanzania remains among 13 countries reporting more than 1000 leprosy cases annually. In 2021, Tanzania detected 1,511 new cases with 10% having grade II disability. Additionally, 14 councils in the country had leprosy rates above 10 cases per 100,000 population. Hence this study aimed at assessing the burden of leprosy and associated factors for disabilities in Tanzania from 2017 to 2020.

**Methodology:** Analytical cross-sectional study was used to investigate all registered treated Leprosy patients from 2017 to 2020. To assess disease burden and risk factor for disability, Leprosy Burden Score (LBS) and logistic regression were used respectively.

**Result:** A total of 6963 leprosy cases were found from 2017 to 2020. Between the years 2017 and 2020, point prevalence new case detection rate remained the same over the study period. Independent risk factors for disability among leprosy cases included male sex (Adjusted Odds Ratio (AOR)=1.45, 95% Confidence Interval (CI) 1.29-1.63), patient age above 55 years (AOR=2.79, 95% CI 1.95-4.01), Multibacillary (MB) leprosy (AOR=1.46, 95% CI 1.15-1.84), previous treatment (AOR=2.11 95% CI 1.65-2.69) and positive Human Immunodeficiency Virus (HIV) status (AOR=1.67 95% CI 1.16-2.41).

**Conclusion:** No significant change in point prevalence and new case detection rate in the country was observed over the past four years. However, various risk factors including age, gender, patient category, type of leprosy and HIV status were found to be associated with leprosy disability. To

combat this, strategies and interventions are needed such as active case finding, post-exposure prophylaxis, provision of MDT and engaging political leaders and key actors in community sensitization, with a focus on endemic areas.

# Introduction

Leprosy is a chronic infectious disease caused by slow growing bacteria called mycobacterium leprae affecting mainly skin, peripheral nerve, eyes and lining of the nasal mucosa with varied clinical presentations (1–3). Transmission of leprosy occurs through infectious droplets and prolonged frequents skin contact with untreated patients (1,4).

Late diagnosis and partial treatment would result into permanent disability and complications including deformities, amyotrophy, sensory loss, wounds on the hands and feet. The aforementioned complication could result into discrimination, social stigma and separation, mental health issues which may contribute to job loss and effect on the economy of the country (5–7).

Globally, leprosy elimination was attained in the year 2000. Afterwards there have been significant decrease in number of cases year by year up to 2005 where majority of countries globally have achieved elimination (8,9). Like other countries, Tanzania attained leprosy elimination status in 2006 (10). Despite the fact that majority of the countries have achieved leprosy elimination, WHO global epidemiological Leprosy update of 2021 showed that there are 13 countries globally (including Tanzania) which are still reporting more than 1000 leprosy cases per year (11).

For the year 2021, 140,594 new leprosy cases detected globally out of which 8,492 (6.0%) had grade II disability and 9,052 (6.4%) were children below the age of 15 years. The trend of leprosy cases notified by World Health Organization (WHO) showed that top three leading region with highest number of new leprosy cases included South East Asia with 93,485 cases followed by Africa with 21,201 cases and America 19,826 cases (11).

In Tanzania, a total of 1,511 new cases were detected in the year 2021, with 154 (10%) having grade II disability and 41 (3%) being children under the age of 15 (11,12). This increase was mainly noted in 14 councils in the country reporting over 10 cases per 100,000 population (12). Additionally, there is a significant diminish in funds to support Leprosy interventions in the country following the declaration of Leprosy elimination.

Without innovative interventions in identified burdened districts, there will be a continuous transmission of disease in the community and the disease will remain to be endemic in those areas (13).

With the country in need to realize Leprosy elimination in all localities, there is a need to formulate strategies which includes active case search, contact tracing, management of cases and associated complications with the aim of stopping the transmission of Leprosy. Additionally, we need to understand the Leprosy trend in this hotspot areas and factors associated with disabilities.

Hence this study described the burden of leprosy trend and risk factors for disabilities among leprosy cases detected in Tanzania from 2017 to 2020. The results obtained will serve as a wake-up call to national and local authorities in enhancing Leprosy Elimination Campaigns (LEC), active case findings, contact tracing, Leprosy Post Exposure Prophylaxis (LPEP) and therapeutic management for Prevention Of Disability (POD) including rehabilitation services.

# Methodology

## Study Design

An analytical cross-sectional study was conducted among leprosy cases who were enrolled on treatment from January 2017 to December 2020.

## Study Area

This study included all 26 regions of Tanzania's mainland and two islands of Zanzibar. In Tanzania, 4,013 of the 8,446 total health facilities are Multidrug Therapy (MDT) centers, which provide management of Leprosy patients as well as ensuring patients take medication as recommended (10).

## Study Population

The study population comprised all Leprosy cases who were enrolled on treatment from January 2017 to December 2020 from National Tuberculosis and Leprosy Program database in all 26 regions in Tanzania mainland and two islands of Zanzibar.

## Dataset description and data abstraction procedure

Leprosy data collection begins at the health facilities. Patients usually attend to the facility on their own after being sick and present with history of skin patches, numbness/weakness of feet’s and

hands, ulcers on hands and feet's (depending on the stage of the disease) where they will pay for registration in order to see a clinician for diagnosis and treatment.

Once the patient is assessed by a clinician and found to meets the criteria of diagnosis by having one or more of the following cardinal signs of leprosy that is hypo-pigmented or reddish lesion with definite loss of skin sensation, enlarged peripheral nerve trunk with muscle weakness and presence of acid fast bacilli in a slit skin smear or microscopy (14), patient will be linked to Tuberculosis and Leprosy clinic where treatment will be initiated by trained dot nurses. Additionally, the patient will be assessed for disability and will be graded as either having grade 0, grade I or grade II disability. Grade 0 disability means absence of disability (no anesthesia) and no visible damage or deformities on eyes, hands and feet. Grade I disability is when the patient is presenting with loss of protective sensibility in the eyes, hands or feet, but no visible damage or deformities whereas grade II disability is the presence of deformities or visible damage to the eyes (lagophthalmos and/or ectropion, trichiasis, corneal opacity, visual acuity less than 0.1 or difficulty counting fingers at 6 meters), visible damage on hands or feet (hand with ulcerations and/or traumatic, resorption, claw, fallen hand, ulcers; feet with trophic and/or traumatic injuries, resorption, claw, foot drop, ulcers, ankle contracture) (14).

Treatment of Leprosy patients according to Tanzania policy its free of charge and patients will be required to undergo treatment for 6 to 12 months.

After diagnosis and initiation of treatment, patient will be recorded in a facility leprosy register and information will be shared to District Tuberculosis and Leprosy Coordinator (DTLC) where patient will be registered as a case based in Electronic Tuberculosis and Leprosy (ETL) System. ETL is web based designed electronic system that acts as surveillance tool for monitoring performance of different national program indicators. The system also monitors progress on treatment of TB, DR TB and Leprosy patients from when registered throughout to treatment completion. Immediately after registration of patients in the system, this information is reflected instantly at all levels (National and Subnational level). Since all patients diagnosed with Leprosy are registered in the database, hence these data approximate incidence of Leprosy in the country. Nevertheless, there is a possibility of small proportion of missed cases not attending the health facilities for treatment.

Data was extracted from the ETL system in form of excel sheet.

## Variables

The dependent variable in this study was Leprosy disability. This was a binary outcome, with the first outcome involving patients with grade 0 disability and the second outcome involving patients with grade I and II disabilities.

Independent variables included age, sex, classification of leprosy, patient category and HIV status.

## Data Analysis

Data extracted from ETL in form of excel was then cleaned and analyzed using Stata version 15.0. Demographic and clinical variables were presented using frequencies and proportions. The regional leprosy burden in the country was determined using LBS developed by WHO (13). Table 1 below narrates each indicator assessed and score guide.

**Table 1: Scale for the leprosy burden score (LBS) assessment**

| **Scale** | **Detection (New cases) for regions** | **Point prevalence rate per 10,000 population** | **New Case Detection Rate per 100,000 population** | **% MB in new Cases** | **% Children in new cases** | **% Female in new cases** | **% Grade II disability in new cases** | **Grade II disability rate per 100,000 population** | **$^{\alpha}$P/D ratio** | **LBS** |
|---|---|---|---|---|---|---|---|---|---|---|
| High | >100=2 | >2=2 | >20=2 | <50=2 | >20=2 | <40=2 | >20=2 | >1=2 | >2=2 | ≥5=2 |
| Medium | 21-100=1 | 1-2=1 | 10-20=1 | 50-75=1 | 10-20=1 | >60=1 | 10-20=1 | 0.5-1=1 | 1-2=1 | 3-4=1 |
| Low | 0-20=0 | <1=0 | <10=0 | 76-100=0 | <10=0 | 40-60=0 | <10=0 | <0.5=0 | <1=0 | 0-2=0 |

$^{\alpha}$P/D: Prevalence/Detection

LBS compose 9 leprosy elimination indicators (13). High = Light yellow (score = 2), Medium = Gray (score = 1), and Low = Light blue (score = 0) were the three grades assigned to each indicator. The LBS was graded into three levels based on the sum of the individual indicator scores: High=Light yellow (Score 2) when the LBS was equal to or greater than 5, Median=Gray (Score=1) when the LBS was between 3 and 4, and Low=Light blue (Score-0) when the LBS was 2 and below. Yearly projected population for respective years for calculation of different indicators was obtained from Tanzania National Bureau of Statistics (NBS). LBS was determined for all 26 regions in Tanzania mainland and two islands of Zanzibar from the year 2017 to 2020 and were categorized as high, medium and low. Map was drawn using QGIS software 3.26.3 to show trend in Tanzania between the year 2017 and 2020. All shapefiles are openly available sources

(https://www.nbs.go.tz/index.php/en/census-surveys/gis/385-2012-phc-shapefiles-level-one-and-two). The shapefiles were made based on the 2012 population and housing census, but in this study, shapefiles have been modified to capture all the regions information.

To ascertain the statistical significance of a trend of leprosy elimination indicators, chi square test for trend was performed and p-value for respective indicators assessed was reported.

In determining risk factors associated with leprosy disability, logistic regression was utilized. Factors such as sex, age group, patient category, classification of leprosy patients and HIV status were all assessed. Factors that were statistically significant at a p-value of $\leq 0.2$ in the bivariate analysis were considered as potential risk factors and included in the multivariable model. Adjusted Odds ratio and their respective 95% confidence intervals were reported.

## Ethical Clearance

The study reviewed secondary data that are part of routine medical and public health surveillance, therefore formal ethical clearance was not required. Approval to use the national TB program data was obtained from Permanent Secretary at the Ministry of Health. Confidentiality of patient data was assured by coding the personal identifiers of patients.

# Result

## Demographic and clinical characteristics of leprosy cases

A total of 6963 leprosy cases were notified between the year 2017 to 2020. The disease was found to be common among male patients 4478 (64.3%). Most reported cases 2,061 (38.7%), were between the age of 35 to 54 years. Majority of the notified cases were new patients 6,661 (95.7%) and were classified as having Multi bacillary leprosy (MB) 6,465 (92.9%). Moreover, assessment conducted before treatment initiation have shown that only 655 (10.4%) of the patients had disability grade II. Table 2 provide detailed information.

**Table 2:** Demographic and clinical characteristics of leprosy patients in Tanzania from 2017 to 2020 (n=6963)

| Variable | Frequency (n) | Percentage (%) |
|---|---|---|
| **Sex** | | |
| Femal[illegible] | 2,485 | 35.7 |
| Male | 4,478 | 64.3 |

| | | |
|---|---|---|
| **Age group (years)** | | |
| ≤14 | 276 | 4.0 |
| 15-34 | 1,929 | 27.7 |
| 35-54 | 2,697 | 38.7 |
| ≥55 | 2,061 | 29.6 |
| **Category of Patient** | | |
| New | 6,661 | 95.7 |
| Previous treated | 302 | 4.3 |
| **Classification** | | |
| Paucibacillary (PB) | 498 | 7.1 |
| Multibacillary (MB) | 6,465 | 92.9 |
| **Disability grading at the start of treatment** | | |
| Grade 0 | 4,337 | 62.3 |
| Grade I | 1,310 | 18.8 |
| Grade II | 655 | 9.4 |
| Not classified | 661 | 9.5 |

## Regional burden of leprosy in Tanzania from the year 2017 to 2020

Over the years 2017 to 2020, the country observed a decline in the point prevalence of Leprosy from 0.32 to 0.25 per 10,000 people. Although there was a 21.9% reduction, it was not statistically significant (P=0.484). Similarly, the New Case Detection Rate (NCDR) showed a decrease from 3.1/100,000 to 2.4/100,000, but the changes were not statistically significant (P=0.488).

Moreover, there was a slight increase in newly reported cases of MB leprosy, rising from 92% to 93.3%, though it did not reach statistical significance (P=0.083). The proportion of children diagnosed with leprosy remained stable at around 4% throughout the four years (P=0.273) and the female proportion among new cases showed a slight decline from 36.7% to 34.8% (P=0.299), though without statistical significance.

Lastly, the percentage of newly diagnosed cases with grade two disabilities saw a slight increase from 8.4% to 9.1%, but it was not statistically significant (P=0.102). Table 3 provide detailed information.

**Table 3:** Trend in Leprosy elimination indicators in Tanzania from 2017 to 2020

| Year | Population Estimate | Registered cases at end of year | New cases detected | Point prevalence per 10,000 population | NCDR per 100,000 population | % MB in new Cases | % Ch[illegible]ren in new cases | % Female in new cases | % G2D in new cases | G2D rate per 100,000 population |
|---|---|---|---|---|---|---|---|---|---|---|
| **`2017** | 51,557,363 | 1648 | 1585 | 0.32 | 3.1 | 92 | 4.1 | 36.7 | 8.4 | 0.28 |
| **2018** | 54,199,163 | 1924 | 1869 | 0.35 | 3.4 | 92.4 | 4.0 | 36.8 | 8.9 | 0.33 |
| **2019** | 55,890,747 | 1929 | 1823 | 0.35 | 3.3 | 93.2 | 3.9 | 34.0 | 9.1 | 0.35 |
| **2020** | 57,637,628 | 1462 | 1384 | 0.25 | 2.4 | 93.3 | 4.0 | 34.8 | 9.1 | 0.24 |
| **Trend test p value** | | | | P=0.484 | P= 0.488 | P=0.083 | P=0.273 | P=0.299 | P=0.102 | P=0.729 |

From 2017 to 2020, eight (8) regions had a more than 50% decrease in the number of leprosy cases reported, with the highest reduction observed in Manyara, Mbeya and Songwe regions with a 100% (P=0.124), 82.6% (P=0.114) and 75% (P=0.685) decrease respectively. However, the observed reduction in number of cases were not found to be statistically significant.

On the other hand, despite four (4) regions reporting an increase in number of leprosy cases for more than 50% between 2017 and 2020, once again, these observed increases were not found to be statistically significant. These regions were Katavi, Mara, Morogoro and Unguja with 3 times (P=0.275), 4 times (P=0.197), 67.5% (P=0.302) and 53.2% (P=0.195) increase respectively. Moreover, there was no significant decrease in number of regions reporting more than 100 new cases per year from 7 regions in 2017 to 3 regions in 2020 (P=0.180).

Although not statistically significant, majority of the regions have achieved a point prevalence of less than 1 per 10,000 population (P=0.815). As for the year 2020, Unguja Island is the only region with a point prevalence of more than 1 per 10,000 population.

Unguja Island has routinely recorded a high rate of newly leprosy cases among children, ranging from 17% in 2017 to 20% in 2020 (P=0.594). However, this difference in incidence among children was not statistically significant. Furthermore, there is no statistical significance increase

in the number of regions reporting more than 20% of new leprosy cases with disability grade II, from 5 regions in 2017 to 7 regions in 2020 (P=0.920).

Using LBS, number of regions with a high leprosy burden has remained relatively constant between 2017 and 2020, whereas 12 regions are highly burdened namely Tanga, Morogoro, Dar es Salaam, Unguja, Tabora, Shinyanga, Lindi, Pemba, Iringa, Rukwa, Mwanza and Kagera. Table 4, Figs 1 and 2 provides detailed information.

## Factors associated with Leprosy disability in Tanzania from the year 2017 to 2020

From January 2017 to December 2020, 6963 records were found in the NTLP database. Among these, 661 (9.5%) were omitted from the analysis of factors associated with disability because they missed disability grading. The final analysis included 6,302 (90.5%) patients (Fig 3).

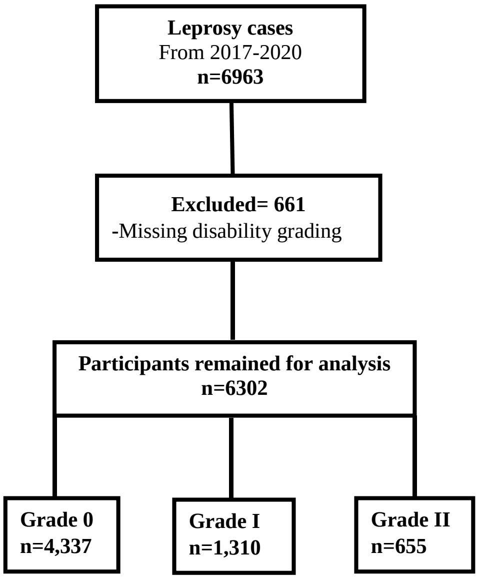


**Fig 3:** Chart outlining how cases were included into the analysis of factors associated with disability among Leprosy cases in Tanzania from the year 2017 to 2020

Information on factors linked to disability is provided in **Table 5.** The multiple logistic regression analysis confirmed the associations between all the variables related to disability in the bivariate analysis. It was observed that male patients were 1.45 more likely to develop disability than female

patients, AOR 1.45 95% CI (1.29-1.63). Patients aged 55 years and above, AOR 2.79 95% CI (1.95-4.01), 15-34 years AOR 2.51 95% CI (1.75-3.60) and 35-54 years AOR 2.32 95% CI (1.62-3.32) were more likely to develop disability compared to those aged below 14 years. Moreover, patients with Multibacillary leprosy were 1.46 times more likely to develop disability than patients with Paucibacillary leprosy, AOR 1.46 95% CI (1.15-1.84). Additionally, previously treated patients were twice as much more likely to develop disability than new patients (AOR 2.12 95% CI 1.66-2.70). Patients with positive HIV status were 1.67 time more likely to have disability than those with a negative HIV status, AOR 1.69 95% CI (1.16-2.41).

**Table 5:** Factors Associated with disability among Leprosy patients registered from January 2017 to December 2020, Tanzania

| Characteristics | Total | Having disability | Univariate analysis | | Multivariate analysis | |
|---|---|---|---|---|---|---|
| | | | COR (95% CI) | P-value | Adjusted OR (95% CI) | P-value |
| **Sex** | | | | | | |
| Female | 2,242 | 585 (26.1) | **Reference** | | **Reference** | |
| Male | 4,060 | 1380 (33.9) | 1.46 (1.30-1.64) | <0.001 | 1.45 (1.29-1.63) | <0.001 |
| **Age group (Years)** | | | | | | |
| ≤14 | 256 | 40 (15.6) | **Reference** | | **Reference** | |
| 15-34 | 1,744 | 556 (31.9) | 2.53 (1.78-3.59) | <0.001 | 2.51 (1.75-3.60) | <0.001 |
| 35-54 | 2,437 | 741 (30.4) | 2.36 (1.67-3.34) | <0.001 | 2.32 (1.62-3.32) | <0.001 |
| ≥55 | 1,865 | 628 (33.7) | 2.74 (1.93-3.89) | <0.001 | 2.79 (1.95-4.01) | <0.001 |
| **Category of Patient** | | | | | | |
| New | 6,020 | 1830 (30.4) | **Reference** | | **Reference** | |
| Previous treated | 282 | 135 (47.9) | 2.10 (1.65-2.67) | <0.001 | 2.11 (1.65-2.69) | <0.001 |
| **Classification** | | | | | | |
| PB | 456 | 105 (23) | **Reference** | | **Reference** | |
| MB | 5,846 | 1860 (31.8) | 1.56 (1.25-1.95) | <0.001 | 1.46 (1.15-1.84) | 0.002 |
| **HIV Status** | | | | | | |
| Negative | 5,444 | 1604 (29.5) | **Reference** | | | |
| Positive | 128 | 51 (39.8) | 1.59 (1.11-2.27) | 0.012 | 1.67 (1.16-2.41) | 0.006 |
| Unknown | 730 | 310 (42.5) | 1.77 (1.51-2.07) | <0.001 | 1.93 (1.65-2.28) | <0.001 |

# Discussion

This study revealed that male sex, older age, multibacillary Leprosy patients, positive HIV status and having history of previous treatment to be independent risk factors for developing disability among Leprosy patients. Additionally, there was no significant change in point prevalence and new case detection rate between the year 2017 and 2020.

## Factors associated with Leprosy disability in Tanzania from the year 2017 to 2020

Findings from our study revealed that there was a strong association between the age of the patient and disability where it was observed that the older the age the higher the risk of having disability. These results were consistent with other studies which affirmed the relationship between age and disability where it was noted that as age increases, the risk also increases (15–20). Possible reasons for increased risk with age could be due to the fact that older individuals face challenges in recognizing early symptoms or seeking medical attention promptly and also older individual experience a longer exposure to risk factors.

Several studies have reported an association between gender and disability risk factors, with males being the most affected (15–17). Our study's findings were consistent with these observations, as we also found that males were more likely to have a disability compared to females. This higher incidence among men may be attributed to specific behavior patterns influenced by social, cultural, and economic factors. Additionally, men tend to exhibit different patterns of health-care seeking behavior compared to women (18).

Moreover, PB patients in our study had less risk of disability compared to MB patients and this result were consistent with other studies which reported that MB patients were strongly linked with disability (15,16,18,19). However, these findings did not correlate with a study conducted in China (17) which reported that majority of PB patients had increased risk of disability.

Similarly, patients who were previously treated were strongly associated with disability compare to newly diagnosed patients. The observed findings was similar to study done in Ethiopia which reported patient who defaulted treatment to have 15 times increased risk of disability compared to new patients (21). Possible reasons for the increased risk of physical disability in leprosy patients

with a history of previous treatment could be the development of drug resistance. This resistance can lead to disease progression, resulting in complications, including nerve damage, and ultimately causing disability.

In the current study it was observed that, HIV positive clients were at increased risk of disability. This could be due to impaired immunity as a result of HIV disease causing Leprosy bacteria to multiply more rapidly and cause more extensive nerve damage, leading to physical disability. Additionally, delay in diagnosis can occur in these patients since HIV-positive individuals may have atypical clinical presentations of leprosy, making diagnosis more challenging. Moreover, both HIV and leprosy can affect the nervous system increasing the severity and speed of nerve impairment, resulting in physical disabilities (22–24).

# Limitations

This study assessed the burden of leprosy using data already present in the national database hence it did not consider patients who were not reported or diagnosed. Additionally, the use of secondary data limited analysis to only variable that were present in the database and some of the risk factors such as occupation, education level, patient delay, nerve lesion could not be assessed. Despite these limitations our study added information on burden of Leprosy and risk factors pertaining to disabilities.

# Conclusion and recommendations

Generally, there has been no significant change in point prevalence and new case detection rate in the country over the past four years. However, various risk factors including age, gender, patient category, type of leprosy and HIV status were found to be associated with leprosy disability. There is a need to develop strategies and interventions that include active case finding, use of new emerging methods like post-exposure prophylaxis, providing Multi-Drug Therapy (MDT) and engaging political leaders in community sensitization efforts, with a specific focus on endemic areas. These initiatives are vital for achieving the goal of eliminating the disease, preventing transmission and reducing disabilities associated with leprosy.

# Acknowledgments

The completion of this study was successful through joint efforts by members from Muhimbili University of Health and Allied Science, Ministry of Health, Tanzania Field Epidemiology and Laboratory Training Program (TFELTP), Centre for Disease Control and Prevention (CDC) through Mzumbe University and different individuals.

Additionally, I would like to express sincere gratitude to the Tanzania National TB and Leprosy Program (NTLP), Epidemiology and Disease Control Section in the Ministry of Health and Tanzania Field Epidemiology and Laboratory Training Program for their support and provision of data for analysis

Furthermore, I am grateful to my supervisor Prof.Gideon Kwesigabo and co-supervisor Dr Ally Hussein for their technical assistance while conducting this study

# Author Contributions

Conceptualization: GWM, AKH, GK, WS

Data Curation: GWM

Formal Analysis: GWM

Investigation: GWM, AKH, GK, WS, AS, VM, DK, EN

Methodology: GWM, AKH, GK, WS

Supervision: AKH, GK, AS, VM, WS, RK, EN, PS, RB, DK

Validation: GWM, AKH, GK, WS, EN, RB, PS, AS, VM, RK

Visualization: GWM, AKH, GK, VM, WS, RB, EN, DK, RK, PS

Writing – Original Draft Preparation: GWM, AKH, GK

Writing – Review & Editing: GWM, AKH, GK, AS, VM, WS, RK, DK, EN, PS, RB

# Reference

1. World Health Organization (WHO). Leprosy. 2022.

2. Hastings RC, Gillis TP, Krahenbuhl JL, Franzblau SG. Leprosy. 1988;1(3):330–48.

3. Han XY, Silva FJ. On the Age of Leprosy. PLoS Negl Trop Dis. 2014;8(2).

4. Lastória JC, de Abreu MAMM. Leprosy: Review of the epidemiological, clinical, and etiopathogenic aspects - Part 1. An Bras Dermatol. 2014;89(2):205–18.

5. Raposo MT, Reis MC, Caminha AV de Q, Heukelbach J, Parker LA, Pastor-Valero M, et al. Grade 2 disabilities in leprosy patients from Brazil: Need for follow-up after completion of multidrug therapy. PLoS Negl Trop Dis. 2018;12(7):1–12.

6. Trautman JR. Bulletin of the New York Academy of Medicine a Brief History of Hansen'S Disease*. 1983;60(7).

7. Berkowitz AL, Raibagkar P, Pritt BS, Mateen FJ. Neurologic manifestations of the neglected tropical diseases. J Neurol Sci. 2015;349(1–2):20–32.

8. World Health Organization (WHO). Global Leprosy strategy 2016–2020, Accelerating towards a leprosy-free world. World Health Organization; 2020. 34 p.

9. World Health Organization. WHO Expert Committee on Leprosy. World Health Organ Tech Rep Ser. 2012;(968).

10. Ministry of Health Tanzania. Report of the United Republic of Tanzania National Tuberculosis and Leprosy Programme External Review. 2020;

11. World Health Organization. Global leprosy (Hansen disease) update, 2021: moving towards interruption of transmission. Wkly Epidemiol Rec. 2022;36:429–50.

12. Tanzania National Tuberculosis and leprosy Programme. Annual Tuberculosis and Leprosy report 2021. 2021;

13. World Health Organization (WHO). Progress towards the reduction of the burden of Leprosy 2000 - 2011. Lepr Elimin Prog Rep 2000-2011. 2011;48.

14. The International Federation of Anti-Leprosy Associations I. How to diagnose and treat Leprosy. 2019;47.

15. Santos VS, Santos de Matos AM, Alves de Oliveira LS, Dolce de Lemos LM, Gurgel RQ, Reis FP, et al. Clinical variables associated with disability in leprosy cases in northeast Brazil. J Infect Dev Ctries. 2015 Mar 2;9(03):232–8.

16. MEIMA A, SAUNDERSON PR, GEBRE S, DESTA K, OORTMARSSEN GJ VAN, HABBEMA JDF. Factors associated with impairments in new leprosy patients: the AMFES cohort. Lepr Rev. 1999;70(2):189–203.

17. Chen X, Liu H, Shui T-J, Zha S. Risk factors for physical disability in patients with leprosy disease in Yunnan, China: Evidence from a retrospective observational study. Nardi SMT, editor. PLoS Negl Trop Dis. 2021 Nov 10;15(11):e0009923.

18. Monteiro LD, Martins-Melo FR, Brito AL, Alencar CH, Heukelbach J. Physical disabilities at diagnosis of leprosy in a hyperendemic area of Brazil: trends and associated factors. Lepr Rev. 2015 Sep 1;86(3):240–50.

19. Srinivas G, Muthuvel T, Lal V, Vaikundanathan K, Schwienhorst-Stich E-M, Kasang C. Risk of disability among adult leprosy cases and determinants of delay in diagnosis in five states of India: A case-control study. Phillips RO, editor. PLoS Negl Trop Dis. 2019 Jun 27;13(6):e0007495.

20. Shumet T, Demissie M, Bekele Y. Prevalence of Disability and Associated Factors among Registered Leprosy Patients in All Africa Tb and Leprosy Rehabilitation and Training Centre (ALERT), Addis Ababa, Ethiopia. Ethiop J Health Sci. 2015 Oct 2;25(4):313.

21. Mulugeta SS, Maseresha BM, Wassihun SG, Moyehodie YA. Risk Factors for Disability Upgrading Among Leprosy Patients During Treatment: Multilevel Modeling Analysis. SAGE Open Nurs. 2022 Jan 9;8:237796082211299.

22. Machado PRL, Machado LM, Shibuya M, Rego J, Johnson WD, Glesby MJ. Viral Co-infection and Leprosy Outcomes: A Cohort Study. Joyce P, editor. PLoS Negl Trop Dis. 2015 Aug 12;9(8):e0003865.

23. Talhari C, Talhari S, Penna GO. Clinical aspects of leprosy. Clin Dermatol. 2015 Jan;33(1):26–37.

24. Deps P, Lucas S, Porro AM, Maeda SM, Tomimori J, Guidella C, et al. Clinical and histological features of leprosy and human immunodeficiency virus co-infection in Brazil. Clin Exp Dermatol. 2013 Jul;38(5):470–7.

# Supporting information

**S1 Table**. Trend in Leprosy elimination indicators by regions from the year 2017 to 2020

**S1 Fig**. Distribution of Leprosy burden in Tanzania Regions for the year 2017

**S1 Fig**. Distribution of Leprosy burden in Tanzania Regions for the year 2020

S1_Table.pdf

Click here to access/download
**Supporting Information**
S1_File.pdf.pdf

S1_fig.tif

Click here to access/download
**Supporting Information**
S1_fig.tif.tif

S2_fig.tif

Click here to access/download
**Supporting Information**
S2_fig.tif.tif
