# Supplementary material for: Burden of leprosy and associated risk factors for disabilities in Tanzania from 2017 to 2020
Source: PLoS One. 2024 Oct 10;19(10):e0311676. doi: 10.1371/journal.pone.0311676 (PMC11466380; doi:10.1371/journal.pone.0311676)
Supplement: Attachment — Submitted filename: Response to reviewers comments.docx [file pone.0311676.s006.docx]

**Dear Editor,**

We express our gratitude to the editor and reviewers for their valuable comments, which have significantly enhanced our manuscript titled **"Burden of Leprosy and associated risk factors for disabilities in Tanzania from 2017 to 2020".** We have taken into account all the comments and integrated their suggestions into the revised version of the manuscript. We believe that we have effectively addressed the raised concerns and that, after incorporating the changes, our manuscript is now appropriate for publication.

Kind regards,

**Dr. George Mrema**

**Editor**

**Comments/concerns/suggestions by editor:**

1. The finding mentioned in the abstract section Line #21 “point prevalence new case detection rate remained the same over the study period.” and the one mentioned in lines #164-165 “Over the years 2017 to 2020, the country observed a decline in the point prevalence of Leprosy 165 from 0.32 to 0.25 per 10,000 people” is not consistent.

**Response to the editor:** We appreciate editor’s observations. We have rephrased the results in abstract section to resemble those in the results section to ensure consistency (line 22-25).

2. Study design: instead of “analytical cross-sectional study’ I suggest ‘A retrospective cross-sectional study’.

**Response to the editor:** We appreciate editor’s suggestions. Study design has been changed from analytical cross-sectional study to retrospective cross-sectional study (line 18 and line 88).

3. I suggest including a map that shows the distribution of leprosy in the country.

**Response to the editor:** We thank the editor for this suggestion. A map showing country distribution of leprosy cases across all regions has been included (line 196-198 and line 201-207).

4. The first paragraph in the discussion section looks like a conclusion you may move it to the conclusion section. I suggest removing the subheading within the discussion.

**Response to the editor:** We appreciate the suggestion. The first paragraph in discussion section has been moved to conclusion (line 358-361).

5. Finding related to point prevalence (burden) is not sufficiently discussed.

**Response to the editor:** We thank the editor for the comment. We have added a paragraph in discussion section that sufficiently discuss the burden of leprosy in the country in relation to other previous studies (line 302-322).

6. Provided that leprosy is a chronic disease with a long incubation period; is it possible to determine the pattern of the disease within 4 years? The conclusion made may be misleading “No significant change in point prevalence and new case detection rate in the country was observed over the past four years”.

**Response to the editor:** We thank the editor for pointing out this. We have added a recommendation that further studies are needed to explore the long-term pattern of disease in the country. Moreover, we have acknowledged in limitation the short duration of our study to be hindrance factor in interpretation of burden (line 35-36, line 350-356, and line 364-365).

**Reviewer No. 1**

**Comments/concerns/suggestions by reviewer 1:**

1. Need for more information on leprosy elimination program activities effectiveness/ineffectiveness in Tanzania that will support recommendations made on the manuscript.

**Response to the reviewer:** We appreciate the reviewer's suggestions. We have added the information on effectiveness/ineffectiveness on leprosy elimination program. Additionally, recommendations has been rephrased (line 32-36, line 67-75, and line 361-365).

2. Table 2: Your data revealed that 64.3% of males compared to 35.7% had leprosy. Did you try to find out any reason for this in the Tanzanian setting? Is there any fundamental difference in sex making the male gender more likely to develop leprosy compared to the female gender? Could it be an equity issue where the female gender has lesser access to leprosy services compared to the male gender?

**Response to the reviewer:** Thank you so much for the comment. We have added a paragraph in discussion section clearly explaining the gender distribution of leprosy cases in relation to other studies. Additionally, we have pointed out the reasons as to why more males in our context (line 285-289).

3. Table 5: The HIV category “Unknown” is 730 with an AOR of 1.93 likely to develop G2D compared to HIV negative. Could including unknown HIV status in the analysis not influence the outcome?

**Response to the reviewer:** We thank the reviewer for pointing out this. We have omitted patients with unknown HIV status in the analysis of risk factor for leprosy disability and reanalysis was conducted (line 259-276) and Table 4).

4. Lines 11, 34-35: mycobacterium leprae is a scientific name and thus should be written in italics with the “M” in capital.

**Response to the reviewer:** We appreciate the reviewer's suggestion. Changes has been made, the scientific name is now written as required in the whole document (line 11 and line 38-39).

5. Line 36: important to precise “nasal droplets” instead of droplets simply.

**Response to the reviewer:** We appreciate the suggestion. The word "droplets" has been changed to "nasal droplets" (line 40).

6. Line 40: Complications instead of complication.

**Response to the reviewer:** We thank the reviewer for the comment. We have change "complication" to "complications" (line 44).

7. Line 47: it is important to precise whether the authors are talking about 1000 new leprosy cases on 1000 leprosy cases. These do not mean the same thing.

**Response to the reviewer:** We thank the reviewer for pointing out this. We have rephrased our statement to remove ambiguity and its now clear for the readers (line 52-53).

8. Lines 49-52: I think that if you are talking about data from a single year (2021 in this cases), you cannot use trends to describe difference in new case detection among WHO regions.

**Response to the reviewer:** Thank you so much for the comment. We have modified our statement to describe the difference in new case detection, the word trend has been removed (line 55-58).

9. Line 55. Were the 10 cases per 100,000 population reported by all the 14 councils put together or by each one of the 14 councils?

**Response to the reviewer:** Thank you for this observation. We have revised statement to be clearer to the reader that all those 14 councils individually report more than 10 cases per 100,000 population (line 60-62).

10. Lines 61-64: The first letter of word leprosy is capitalized within sentences. Is there any particular reason for this?

**Response to the reviewer:** We appreciate for this observation. We have checked and changed the unnecessary capitalization of the word leprosy throughout the entire reviewed manuscript.

11. Line 92: dot is an abbreviation for “directly observed treatment” and should thus be written in capital and the full meaning given in bracket here where it has been used for the first time

**Response to the reviewer:** Thank you so much for the comment. The word DOT is now written in capital and it has been written in full with the abbreviations in bracket. Additionally, we have added a definition of DOT to make sure all readers get the meaning of it (line 109-111).

12. Line 102: its should be replaced with is.

**Response to the reviewer:** We appreciate the suggestion. The word "its" has been changed to "is" (line 120).

13. Line 150: Do you mean the National TB and Leprosy Program or TB Program?

**Response to the reviewer:** Thank you so much for the comment. We meant National TB and Leprosy Program (NTLP) (line 170-171).

14. Line 159 and Table 2: There is a discrepancy in the proportion of G2D cases reported: 10.4% vs 9.4%.

**Response to the reviewer:** Thank you so much for the observation. We have corrected the discrepancy of data that was in description and the one in the table to match each other (line 195 and Table 2).

15. Lines 185-186: Reporting of increases in number of leprosy cases should be consistent with for all 4 regions mentioned. Use either percentage increase or point increase for all and not a mixture of percentage and point increase.

**Response to the reviewer:** We appreciate the suggestion. We have maintained consistency in reporting number of leprosy cases by using percentage increase in the whole description (line 233-236).

16. Lines 214-215: regarding age and disability, persons aged 15 and over (usually considered as adults in leprosy) were all likely to develop disability (AOR 2.32 – 2.79) compared to those 14 years of age and below (usually considered as children in leprosy). I think it would be better categorizing age into two categories: children (<=14 yrs) and adults (>=15yrs) and redo the analysis.

**Response to the reviewer:** We appreciate the reviewer's suggestion. We have recategorized the age group as suggested and we have repeated the analysis (Table 2, line 271-273 and Table 4).

**Reviewer No. 2**

**Comments/concerns/suggestions by reviewer 2:**

1. Line 15: To assess the burden of leprosy and disability of Tanzania during the period.... and to describe/determine its risk factors.

**Response to the reviewer:** We appreciate the suggestion. We have rephrased our statement as suggested and it now written “Hence, this study aimed to assess the burden of leprosy and disability in Tanzania from 2017 to 2020 and to determine the associated risk factors” (line 16-17 and line 80-81).

2. Line 18: Month start to end?

**Response to the reviewer:** Thank you so much for the comment. We have in cooperated the start month and end month in suggested area (line 19 and line 89).

3. Line 19: Conditional LR or other?

**Response to the reviewer:** We thank the reviewer for the comment. This was not a conditional logistic regression since it was a cross sectional study. Moreover, we have added type of logistic regression to improve clarity (line 20 and line 163).

4. Table 2: Gender

**Response to the reviewer:** We appreciate the suggestion. The word "sex" has been changed to "gender" (Table 2).

5. Table 3: Child Disability rate?

**Response to the reviewer:** Thank you so much for the comment. No, the percentage recorded represents the proportion of children within the newly diagnosed cases (Table 3).
