# Supplementary material for: Burden of leprosy and associated risk factors for disabilities in Tanzania from 2017 to 2020
Source: PLoS One. 2024 Oct 10;19(10):e0311676. doi: 10.1371/journal.pone.0311676 (PMC11466380; doi:10.1371/journal.pone.0311676)
Supplement: Attachment — Submitted filename: General remarks on Revision 1.docx [file pone.0311676.s007.docx]

General remarks,

Most of the queries raised in the first review has been taken care of by the authors in Revision 1 of the manuscript.

I understand from information in lines 100 – 103, that leprosy detection in Tanzania is largely passive, with no major efforts by the National TB and Leprosy Control Programme to improve upon cases detection.

With this background, and with the graphical presentation of data (from Table 3 in the manuscript) below, could the authors make some clarifications on the increase of leprosy prevalence and case detection of 17% and 20% respectively from 2017 to 2018, followed by the remarkable decrease in by 24% between 2019 and 2020.

I feel that there are programmatic issues related to these trends that require elucidation.

Trend in leprosy prevalence and new case detection from 2017 to 2020 in Tanzania

Rate/10,000 population

Number of leprosy cases

|  | Reg Prev | NCD | Prev. Rate/ 10,000 | NCDR/ 10,000 |
| --- | --- | --- | --- | --- |
| 2017 | 1648 | 1585 | 0.319644 | 0.307425 |
| 2018 | 1924 | 1896 | 0.354987 | 0.349821 |
| 2019 | 1929 | 1823 | 0.345138 | 0.326172 |
| 2020 | 1462 | 1384 | 0.253654 | 0.240121 |

| Trends | Registered Prevalence | | New case detection | |
| --- | --- | --- | --- | --- |
|  | Absolute | Relative | Absolute | Relative |
| Increase from 2017-2018 | 276 | 17% | 311 | 20% |
| Decrease from 2019-2020 | 467 | 24% | 439 | 24% |
