# Supplementary material for: Burden of leprosy and associated risk factors for disabilities in Tanzania from 2017 to 2020
Source: PLoS One. 2024 Oct 10;19(10):e0311676. doi: 10.1371/journal.pone.0311676 (PMC11466380; doi:10.1371/journal.pone.0311676)
Supplement: Attachment — Submitted filename: Response to Reviewers.docx [file pone.0311676.s008.docx]

**Dear Editor,**

We appreciate all reviewers for their valuable comments, which have greatly enhanced the quality and content of our manuscript titled **"Burden of Leprosy and associated risk factors for disabilities in Tanzania from 2017 to 2020".** We have taken into account all the comments and integrated their suggestions into the revised version of the manuscript. We believe that we have effectively addressed the raised concerns and that, after incorporating the changes, our manuscript is now appropriate for publication.

Kind regards,

**Dr. George Mrema**

**Reviewer No. 1**

**Comments/concerns/suggestions by reviewer 1:**

1. I understand from information in lines 100 – 103, that leprosy detection in Tanzania is largely passive, with no major efforts by the National TB and Leprosy Control Programme to improve upon cases detection. With this background, and with the graphical presentation of data (from Table 3 in the manuscript) below, could the authors make some clarifications on the increase of leprosy prevalence and case detection of 17% and 20% respectively from 2017 to 2018, followed by the remarkable decrease in by 24% between 2019 and 2020. I feel that there are programmatic issues related to these trends that require elucidation.

**Response to the reviewer:** We appreciate the reviewer's observation. We have improved the information in the discussion part elaborating the reason for fluctuation in both NCDR and prevalence (line 307-314).

**Reviewer No. 2**

**Comments/concerns/suggestions by reviewer 2:**

1. With an objective to assess risk factor for disability, the study should have used primary data also and investigated the logical risk factors (lepra reactions, time delay from symptom noticed to diagnosis), etc.

**Response to the reviewer:** We are grateful for your feedback. While primary data collection could have provided additional insight into specific risk factors such as lepra reactions and time delays from symptom noticed to diagnosis, the use of secondary data in our setting still allowed for a comprehensive analysis of the associated risk factors for disabilities since the information used contained variables collected from registration of patients who were registered for treatment. Additionally, we acknowledged this as a limitation and added a recommendation for further research to explore more risk factors (line 36, line 361-363 and line 372-373).
