# Supplementary material for: Burden of leprosy and associated risk factors for disabilities in Tanzania from 2017 to 2020
Source: PLoS One. 2024 Oct 10;19(10):e0311676. doi: 10.1371/journal.pone.0311676 (PMC11466380; doi:10.1371/journal.pone.0311676)
Supplement: Attachment — Submitted filename: Response to Reviewers.docx [file pone.0311676.s009.docx]

**Dear Editor,**

We thank the reviewer for the additional comments, which continue to improve the quality and content of our manuscript titled **"Burden of Leprosy and associated risk factors for disabilities in Tanzania from 2017 to 2020".** We have addressed the comments and integrated their suggestions into the revised version of the manuscript. We believe that we have effectively addressed the raised concerns and that, after incorporating the changes, our manuscript is now appropriate for publication.

Kind regards,

**Dr. George Mrema**

**Reviewer No. 2**

**Comments/concerns/suggestions by reviewer 2:**

1. The nature of data allows only this level of analysis; this may be mentioned as a limitation of the study.

**Response to the reviewer:** In the limitations section, we mentioned that we relied on secondary data, which comes with its own limitations. We also suggested that additional research is necessary to investigate more risk factors (line 35-36, line 349-351 and line 359-361).

**Reviewer No. 3**

**Comments/concerns/suggestions by reviewer 3:**

1. I still think some contextual and programmatic information are needed to explain the high leprosy burden 14 counties: either real high burden or because of availability of quality services, trained health care workers and less stigma in these counties compared to others. This information is necessary to support a stratified and targeted intervention as recommended by the author.

**Response to the reviewer:** We are grateful for your comment. We have added information in the background explaining the reasons behind high burden in 14 districts in the country (line 62-66).
