# Supplementary material for: Burden of leprosy and associated risk factors for disabilities in Tanzania from 2017 to 2020
Source: PLoS One. 2024 Oct 10;19(10):e0311676. doi: 10.1371/journal.pone.0311676 (PMC11466380; doi:10.1371/journal.pone.0311676)
Supplement: Attachment — Submitted filename: Response to Reviewers.docx [file pone.0311676.s010.docx]

**Dear Editor,**

We are grateful to the reviewers for their additional comments, which further improve the quality of our manuscript titled **"Burden of Leprosy and associated risk factors for disabilities in Tanzania from 2017 to 2020".** We have taken the comments into account and incorporated their suggestions into the revised manuscript. We believe we have effectively addressed the concerns raised, and after making these changes, our manuscript is now suitable for publication.

Kind regards,

**Dr. George Mrema**

**Reviewer No. 3**

**Comments/concerns/suggestions by reviewer 3:**

1. Some level of English editing is still needed for the manuscript, the author should leverage the services of professional editors which can also help reduce redundancy in some of the sentences.

**Response to the reviewer:** We are grateful for your comment. The language has been edited, and redundant sentences have been removed by carefully reviewing our manuscript and rephrasing where necessary. This process has improved clarity and conciseness while preserving the intended meaning and essential details of the original text.
